# Acceptance of the Use of Artificial Intelligence in Medicine Among Japan’s Doctors and the Public: A Questionnaire Survey

**DOI:** 10.2196/24680

**Published:** 2022-03-16

**Authors:** Honoka Tamori, Hiroko Yamashina, Masami Mukai, Yasuhiro Morii, Teppei Suzuki, Katsuhiko Ogasawara

**Affiliations:** 1 Graduate School of Health Sciences Hokkaido University Sapporo Japan; 2 Faculty of Health Sciences Hokkaido University Sapporo Japan; 3 Fukushima Medical University Fukushima Japan; 4 Division of Medical Informatics National Cancer Center Hospital Chuo Japan; 5 Center for Outcomes Research and Economic Evaluation for Health National Institute of Public Health Wako Japan; 6 Hokkaido University of Education Iwamizawa Campus Iwamizawa Japan

**Keywords:** artificial intelligence, technology acceptance, surveys and questionnaires, doctors vs public

## Abstract

**Background:**

The use of artificial intelligence (AI) in the medical industry promises many benefits, so AI has been introduced to medical practice primarily in developed countries. In Japan, the government is preparing for the rollout of AI in the medical industry. This rollout depends on doctors and the public accepting the technology. Therefore it is necessary to consider acceptance among doctors and among the public. However, little is known about the acceptance of AI in medicine in Japan.

**Objective:**

This study aimed to obtain detailed data on the acceptance of AI in medicine by comparing the acceptance among Japanese doctors with that among the Japanese public.

**Methods:**

We conducted an online survey, and the responses of doctors and members of the public were compared. AI in medicine was defined as the use of AI to determine diagnosis and treatment without requiring a doctor. A questionnaire was prepared referred to as the unified theory of acceptance and use of technology, a model of behavior toward new technologies. It comprises 20 items, and each item was rated on a five-point scale. Using this questionnaire, we conducted an online survey in 2018 among 399 doctors and 600 members of the public. The sample-wide responses were analyzed, and then the responses of the doctors were compared with those of the public using *t* tests.

**Results:**

Regarding the sample-wide responses (N=999), 653 (65.4%) of the respondents believed, in the future, AI in medicine would be necessary, whereas only 447 (44.7%) expressed an intention to use AI-driven medicine. Additionally, 730 (73.1%) believed that regulatory legislation was necessary, and 734 (73.5%) were concerned about where accountability lies. Regarding the comparison between doctors and the public, doctors (mean 3.43, SD 1.00) were more likely than members of the public (mean 3.23, SD 0.92) to express intention to use AI-driven medicine (*P*<.001), suggesting that optimism about AI in medicine is greater among doctors compared to the public.

**Conclusions:**

Many of the respondents were optimistic about the role of AI in medicine. However, when asked whether they would like to use AI-driven medicine, they tended to give a negative response. This trend suggests that concerns about the lack of regulation and about accountability hindered acceptance. Additionally, the results revealed that doctors were more enthusiastic than members of the public regarding AI-driven medicine. For the successful implementation of AI in medicine, it would be necessary to inform the public and doctors about the relevant laws and to take measures to remove their concerns about them.

## Introduction

### Background

The use of artificial intelligence (AI) in the medical industry promises many benefits. For example, it can yield new diagnostic and therapeutic methods, provide the groundwork for introducing cutting-edge medical technology, and reduce the workload of doctors and care workers [1-3]. AI has been introduced to medicine primarily in developed countries [4]. The United States made an early start in this respect. In April 2018, the country’s Food and Drug Administration (FDA) authorized the first medical device to use AI. The device, named IDx-DR, detects greater than a mild level of the eye disease diabetic retinopathy in patients with diabetes. As the FDA states, IDx-DR provides a screening decision “without the need for a clinician to also interpret the image or results” [5]. Like the United States, Japan wants to drive forward the use of AI in medicine. The country’s Ministry of Health, Labour, and Welfare (MHLW) has designated six health care fields where AI is to be developed. Under the MHLW’s plan, AI will be rolled out relatively early in four of these fields (genome medicine, diagnostic imaging, diagnosis and treatment, and drug development) and then in a more phased manner in the remaining two (long-term care and dementia, surgery) [1]. Despite the MHLW’s efforts, however, Japan lags other developed countries in rolling out AI in health care.

As the shift toward AI in medicine continues apace, there is an urgent need to consider the ethical, legal, and social issues (ELSIs) of this trend [6]. For example, insofar as clinical data are used to develop AI applications, an issue arises regarding patients’ personal data [7]. This issue has not escaped the attention of Japan; in June 2018, the MHLW released an announcement on AI-guided diagnosis, stating that AI will only ever assist a human doctor in forming a final diagnosis and that no matter how advanced AI becomes, decision-making responsibility will always lie with the human doctor [8]. Despite such reassurances, many members of the public remain concerned about where accountability lies in AI-driven medicine. Such misgivings may hinder the rollout of AI in medicine.

A new application can only fulfill its potential if people use it. Exemplifying this principle are South Korea’s mobile electronic medical records (EMRs) [9]. Mobile EMRs are effective for streamlining medical work and minimizing hospital costs, but staff feel strongly disinclined to use them, and the uptake rate is low; this is because the functions are poorly tailored to the user’s needs. This situation demonstrates, according to Kim [9], that an application can only fulfill its true potential if the developers consider user feedback. The success of a technology rollout depends on the technology’s features, but it also depends on popular trends and the broader sociocultural milieu. Toward ensuring successful rollout, trends in public acceptance of the technology and the determinants of such would need to be identified [10,11]. When it comes to AI in medicine, there may be a gap in acceptance between the doctors, who would actually use the AI, and the public, who would receive AI-driven medical services. It is, therefore, necessary to consider acceptance among doctors and among the public. However, in Japan, the acceptance of AI in medicine has not been investigated.

Therefore, the purpose of this study was to obtain detailed data on the acceptance of AI in medicine by comparing the acceptance among Japanese doctors with that among the Japanese public.

### Theoretical Background

In recent years, AI has been developed in the medical industry. For example, AI can detect pulmonary nodules, tuberculosis, and pneumonia in chest radiographs, detect and quantify pulmonary nodules in chest computed tomography (CT) [12], detect suspected large vessel occlusion strokes based on CT images [13], and screen for breast cancer [14].

The technology acceptance model (TAM) is used to investigate the acceptance of new technologies. TAM is a model that explains the process by which users accept and use information systems. There are many extended models, among which the unified theory of acceptance and use of technology (UTAUT), proposed by Venkatesh et al [15] by integrating eight models, explains 70% of the variance in individual intention to use technology while the existing technology acceptance models explain 40%. In UTAUT, the user's intention to use an information system and subsequent use is explained by four components (performance expectancy, effort expectancy, social influence, and facilitating conditions).

### Literature Reviews

Oh et al [16] found that 83.4% of respondents considered that AI would be useful in the medical field, indicating that doctors have a positive attitude toward AI in medicine in a survey of doctors and medical students in Korea. Jonmarker et al [17] investigated participants' confidence in the introduction of AI into a breast cancer screening program in Sweden. Participants in a breast cancer screening program trusted the computer-aided decision-making of their doctors the most. Jutzi et al [18] also conducted a survey of patients with and without a diagnosis of melanoma to investigate the acceptance of AI for melanoma diagnostics in Germany. The results showed that only 41% agreed with the use of AI as a stand-alone system, and 94% agreed with the use of AI as a support system for doctors.

In Japan, a number of studies have polled attitudes regarding the rollout of AI in health care. In one survey by the Ministry of Internal Affairs and Communications (MIC), 81.5% of the polled experts said that they would welcome the use of AI in analyzing biometrics, lifestyle, disease history, genetic data, and other factors to detect precisely symptoms of health conditions or the onset of disease [19]. Another attitudes survey on AI in health care was conducted by Ema et al [20]. In the survey, the respondents agreed strongly with the idea of entrusting AI with driving, disaster management, military matters, and other functions where the rollout of AI requires institutional and social consent. However, they felt that humans should remain the primary actor in matters involving individual choice, such as health management and important life decisions. As insightful as its findings are, the study had examined participants’ views on the use of AI in a number of fields, not only health care. A focus on AI in medicine would present a more detailed picture of the public and expert trust in such. One study that did so was Yokoi and Nakayachi [21]; the study reported that sharing treatment plans resulted in higher trust toward AI but also that this effect was modest. However, they are investigating the reliability of AI in medicine, and there has been no investigation of acceptance focused on AI in medicine in Japan. For the successful implementation of AI in medicine in Japan, it is necessary to investigate not only the reliability but also the acceptance and factors related to acceptance.

In addition, although clarifying the differences in acceptance of AI in medicine between doctors and the public would allow us to consider approaches suitable for each of them in promoting the introduction of AI, most previous studies have focused on either doctors or the public (patients).

Our research question was to what extent AI in medicine has been accepted in Japan and whether there are differences in the acceptance of AI in medicine between doctors and the public.

## Methods

### Survey

For the purposes of the study, AI in medicine was defined as the use of AI to determine diagnosis and treatment without requiring a doctor.

The survey questions were divided into two sections. The first section consisted of items on the respondents’ general attributes, such as sex and whether the respondent was a doctor.

The second section measured the respondents’ acceptance of AI in medicine with questions referred to the UTAUT [15]. Generally, a survey is conducted for people who directly use the system, assuming a specific system in UTAUT. However, in this study, the respondents include the public who does not use the system directly. Also, we assume no specific systems because the AI in medicine was not widespread at the time the survey was conducted in Japan, and the only description of AI was “the use of AI to determine diagnosis and treatment without requiring a doctor” in the questionnaire. Therefore, we modified the questions through discussion to make them suitable for this study. Specifically, questions that are difficult to answer without assuming a specific system were deleted, and we added alternative items for four key components. In addition, since attitude and uneasiness have been verified as a factor influencing intention to use in previous studies, we added a question on attitude and uneasiness in this study [15,22]. Then, we thought it would be difficult to analyze the data by fitting the UTAUT model because we modified the questionnaire for this study, so we measured one element for all items.

There were 20 such items (Textbox 1), each representing a factor of acceptance. Each item was rated on a five-point scale (1 = completely false, 2 = somewhat false, 3 = cannot say either way, 4 = somewhat true, 5 = completely true). A question item about medical costs was rated on a different five-point scale, where 1 = costs will decrease, and 5 = costs will increase.

**Question items measuring acceptance factors**

**Usefulness:** Do you think that AI in medicine will be useful?

**Efficiency:** If AI is used in medicine, do you think doctors could provide services more efficiently?

**Better medical services:** Would using AI in medicine lead to better medical services?

**Mastery:** Could doctors quickly master the use of AI in medicine?

**User-friendliness:** Could doctors easily operate AI in medicine settings?

**Expectations of others:** Do you think people around you are optimistic about the potential of AI in medicine?

**Expectations among patients:** Do you think patients are optimistic about the potential of AI in medicine?

**Brand impact:** Are your views on AI in medicine shaped by the businesses (or brands of such businesses) that developed the AI for medicine?

**Knowledge of AI in other contexts:** Do you know much about the use of AI in contexts other than medicine?

**Knowledge of AI in medicine:** Do you know much about the use of AI in medicine?

**Medical costs:** How do you think AI in medicine will affect medical costs?

**Necessity of legislation:** Do you think the use of AI in medicine should be regulated by legislation?

**General impression:** Do you have a generally favorable impression of the use of AI in medicine?

**Interest in topic:** Are you interested in the topic of AI in medicine?

**Accuracy:** Do you think AI in medicine will deliver accurate diagnoses?

**Concern about data leakage:** Are you concerned that using AI in medicine might lead to the leakage of personal data?

**Concern about accountability:** Are you concerned about who would be accountable for any accident resulting from the use of AI in medicine?

**Intention to use:** Would you be willing to use AI-driven medicine?

**Relevance to life:** Do you think that AI in medicine will play an important role in your life in the future?

**Necessity in medicine:** Do you think that AI will be essential in medicine in the future?

The survey was conducted over three days, from November 13 to 15, 2018. The authors did not obtain Institutional Review Board approval for this study because we used Rakuten Insight to conduct the survey, and we did not obtain any personal information from the respondents. In Japan, researchers do not have to obtain Institutional Review Board approval when subjects can voluntarily decide to participate in a study, there is no intervention in the collection of data, and individuals cannot be identified from the collected data [23]. Respondents were told the length of time to answer the questions, the purpose of the survey, and who conducted the survey on the screen just before they started to answer. Respondents were allowed to stop answering at any time until they answered all the questions. We took the completed responses as respondents’ consent to participate in the survey and used the responses for analysis. In Multimedia Appendix 1, each item in the Checklist for Reporting Results of Internet E-Surveys (CHERRIES) is shown [24].

After the questionnaire survey, the reliability of the questionnaire was examined. First, Cronbach alpha was calculated to confirm the reliability of the entire questionnaire (Cronbach α=.88). Cronbach α values of .70 to .80 or more are regarded as satisfactory [25], the questionnaire of this study was considered to be reliable.

The public and doctor responses for the 20 items were compared using a *t* test. We further calculated Cohen *d* for each item. In general, Cohen *d* of 0.2 is considered small, 0.5 is considered medium, and 0.8 is considered large [26]. In addition, the opposite scale was inverted so that a positive response to the use of AI was 5 and a negative response was 1 (eg, concern about data leakage). The total mean score of the whole scale was calculated for doctors and the public respectively and compared using a *t* test. The statistics were processed using R (version 3.5.1; R Core Team).

### Respondents

An online survey was conducted among the public and among doctors. The sample representing the public survey consisted of 600 individuals across six age cohorts, each of which included 50 men and 50 women. The cohorts were aged 15 to 24, 25 to 34, 35 to 44, 45 to 54, 55 to 64, and 65 years and older. The sample representing doctors consisted of 400 individuals aged 25 years or older. Of these, 350 were men and 50 were women. One doctor was excluded from analysis because he answered that his highest education attainment was “vocational school/junior college,” though doctors needed university education to get a license.

Regarding the respondents’ general attributes, Table 1 shows the sex and age, and Table 2 shows the educational attainment of the doctors and members of the public.

**Table 1:** Sex and age distribution.

Age (years)	Doctors				Public		
	Male	Female	Total	Male		Female	Total
15–24	N/A^a^	N/A	N/A	50		50	100
25–34	7	13	20	50		50	100
35–44	42	19	61	50		50	100
45–54	123	11	134	50		50	100
55–64	141	5	147	50		50	100
65 or older	36	2	38	50		50	100
Total	349	50	399	300		300	600

^a^N/A: not applicable.

**Table 2:** Highest educational attainment.

Highest educational attainment	Doctors	Public
Junior high school	0	8
High school	0	177
Vocational school/junior college	(1)^a^	127
University	398	282
Other	1	6
Total	399	600

^a^One doctor was excluded from analysis because doctors needed university education to get a license.

## Results

Table 3 shows the sample-wide results of 999 respondents for the 20 items on acceptance of AI in medicine. For the following items, under 20% of the responses were negative (“completely false” or “somewhat false”), and over 50% were positive (“completely true” or “somewhat true”): usefulness, efficiency, better medical services, expectations of others, expectations among patients, general impression, relevance to life, and necessity in medicine. The most positively rated of these items were “usefulness” and “necessity in medicine,” the number of positive responses for which were 657 (65.8%) and 653 (65.4%), respectively. One item with a relatively low positive response rate was “intention to use”; only 447 (44.7%) of the respondents indicated that they were willing to use AI-driven medicine (“I would be moderately willing to” or “I would be very willing to”).

Regarding the necessity of legislation and whether there is concern about accountability, only 360 (36.0%) and 315 (31.5%) of the respondents gave a strong affirmative response, respectively. However, when the strong and moderate responses were combined, as many as 730 (73.1%) and 734 (73.5%) gave an affirmative response, respectively.

**Table 3:** Sample-wide results for factors of acceptance.

Items	Mean (SD)
Usefulness	3.66 (0.91)
Efficiency	3.47 (0.89)
Better medical services	3.66 (0.97)
Mastery	3.02 (0.91)
User-friendliness	2.97 (0.91)
Expectations of others	3.44 (0.92)
Expectations among patients	3.48 (0.99)
Brand impact	3.05 (1.07)
Knowledge of AI in other contexts	2.82 (1.04)
Knowledge of AI in medicine	2.34 (1.01)
Medical costs	2.88 (0.99)
Necessity of legislation	3.99 (0.88)
General impression	3.64 (1.00)
Interest in topic	3.52 (0.81)
Accuracy	3.08 (1.10)
Concern about data leakage	3.30 (1.00)
Concern about accountability	3.92 (0.96)
Intention to use	3.31 (0.91)
Relevance to life	3.64 (0.92)
Necessity in medicine	3.70 (0.42)

Table 4 shows the comparative results for the doctors and the public. Responses for eight of the items exhibited a significant intergroup difference at the 5% level of significance: better medical services, mastery, expectations of others, knowledge of AI in medicine, medical costs, interest in the topic, concerns about data leakage, and intention to use. Intergroup difference was particularly notable in “expectations of others” and “intention to use.” Among the public, the median response for both items was neutral (3. “cannot say either way).” Among doctors, the median was a moderately affirmative response (4. “I moderately agree that people around me are optimistic about AI in health care” for the former, and 4. “I would like to use AI-driven medicine in the future” for the latter). Another notable item was “knowledge of AI in medicine.” The median response for this item among doctors was neutral, whereas that among the public was moderately negative (2. “I don’t know all that much about it”). In addition, Cohen *d* for mastery, expectations of others, medical costs, intention to use was small, and Cohen *d* for knowledge of AI in medicine was medium.

**Table 4:** Comparison between doctors and the public regarding factors associated with acceptance.

Items	Doctor			Public			Cohen *d*		95% CI		*P* value
	Median	Mean (SD)	Median		Mean (SD)						
Usefulness	4	3.72 (0.93)	4		3.62 (0.90)	0.11		–0.02 to 0.21		.09	
Efficiency	4	3.44 (0.89)	4		3.50 (0.86)	–0.07		–0.17 to 0.05		.30	
Better medical services	4	3.73 (0.85)	4		3.61 (0.91)	0.15		0.02 to 0.24		.02	
Mastery	3	3.15 (0.95)	3		2.93 (0.97)	0.23		0.10 to 0.34		<.001	
User-friendliness	3	2.97 (0.91)	3		2.97 (0.90)	–0.001		–0.12 to 0.11		.98	
Expectations of others	4	3.56 (0.88)	3		3.37 (0.92)	0.21		0.08 to 0.30		.001	
Expectations among patients	4	3.50 (0.89)	4		3.46 (0.94)	0.05		–0.07 to 0.16		.46	
Brand impact	3	3.04 (0.98)	3		3.06 (0.99)	–0.02		–0.14 to 0.11		.82	
Knowledge of AI^a^ in other contexts	3	2.88 (1.00)	3		2.78 (1.11)	0.10		–0.03 to 0.24		.13	
Knowledge of AI in medicine	3	2.66 (1.00)	2		2.12 (1.01)	0.54		0.41 to 0.67		<.001	
Medical costs	3	3.01 (0.93)	3		2.79 (1.04)	–0.23		–0.35 to –0.10		<.001	
Necessity of legislation	4	4.01 (1.01)	4		3.99 (0.97)	0.02		–0.11 to 0.15		.76	
General impression	4	3.69 (0.85)	4		3.60 (0.89)	0.10		–0.02 to 0.20		.13	
Interest in topic	4	3.61 (1.01)	4		3.46 (0.99)	0.15		0.02 to 0.28		.02	
Accuracy	3	3.11 (0.84)	3		3.06 (0.79)	0.07		–0.05 to 0.16		.32	
Concern about data leakage	3	3.38 (1.08)	3		3.24 (1.11)	0.13		–0.28 to –0.002		.046	
Concern about accountability	4	3.91 (1.01)	4		3.93 (0.99)	0.015		–0.11 to 0.14		.81	
Intention to use	4	3.43 (1.00)	3		3.23 (0.92)	0.21		0.07 to 0.32		.002	
Relevance to life	4	3.62 (0.90)	4		3.66 (0.92)	–0.04		–0.16 to 0.08		.49	
Necessity in medicine	4	3.74 (0.84)	4		3.67 (0.97)	0.07		–0.05 to 0.18		.24	

^a^AI: artificial intelligence.

The total mean score of the whole scale was 65.5 (SD 10.4) for doctors and 64.1 (SD 10.6) for the public, with a difference of 1.4 (95% CI 0.10-2.77). Cohen *d* was 0.14.

## Discussion

### Principal Results

Regarding the sample-wide results, the respondents were generally receptive toward AI in medicine. Particularly respondents have confidence in AI’s usefulness and a belief in its future necessity in medicine. In the MIC survey [19], experts expressed optimism on the use of AI in diagnosis and other aspects of health care. This study revealed that optimism on AI in health care is present among the public and doctors alike, implying that such optimism is broadly held.

Despite their tendency to see AI as useful and necessary in medicine, the respondents were less enthusiastic about the prospect of actually using AI-driven medicine, with only 44.8% of the respondents giving a moderate or strong affirmative response for intention to use. According to a previous study, 41% of respondents in Germany were in favor of using AI alone to diagnose melanoma [18]. In Sweden, 38% of participants in a breast cancer screening program preferred computer-only reading [17]. Furthermore, 35.4% of Korean doctors agreed that AI could replace them in their jobs [16]. The acceptance of AI-driven medicine in Japan seems to be generally consistent with previous studies. The UTAUT model assumes that some factors encourage acceptance of technology, whereas other factors hinder such [22]. The presence of a hindering factor may be the reason that belief in AI’s usefulness and necessity in medicine did not translate directly into a desire to use AI-driven medicine personally.

The majority of the sample expressed a moderate or strong concern regarding the issues of regulatory legislation and accountability. About half of the respondents expressed moderate or strong concern about data leakage. These three items describe ELSIs, which require solutions from a policy perspective. Given that so many of the respondents were concerned about these ELSIs, the ELSIs in question are likely major determinants of acceptance for both doctors and members of the public. In particular, the issue of accountability attracted concern from as many as three-fourths of the respondents, despite the MHLW’s attempts to reassure people that human doctors will always be responsible for the final diagnosis. The causes of such uncertainty are unclear from this study’s results; further research is necessary to identify the causes and derive ways to alleviate the concerns among doctors and the public.

Discussed below is the comparison between doctors and the public. The results revealed significant intergroup differences in eight items. One such difference was in “intention to use”; doctors were more enthusiastic than the public about using AI-driven medicine in the future. Ema et al [20] surveyed the public and 10 other stakeholders on the use of AI in eight areas, one of which was health management. In all eight areas, the study found the public to be more likely than the other stakeholders to answer that humans should remain in control. However, any comparison with this study requires some qualifications owing to salient differences. Ema et al [20] did not include doctors among the ten stakeholders and used a definition of AI different from that used in this study. Nonetheless, Ema et al’s [20] observation that experts are more willing to trust AI than members of the public echoes this study’s observation that doctors, compared with the public, were more receptive to the idea of using AI.

Doctors’ comparative enthusiasm for using AI may be related to the fact that they were also more likely than members of the public to give positive responses to better medical services, mastery, expectations of others, and interest in the topic. That is, the doctors’ intention to use AI may have been motivated by their greater expectations (compared with those held by members of the public) about the potential of AI in medicine. Additionally, members of the public were more likely than doctors to indicate a lack of knowledge about AI in medicine. The fact that members of the public tended to be rather uninformed about AI in medicine may have contributed to their weak (compared with doctors) intention to use AI.

Meanwhile, the responses to the items on medical costs and concern about data leaks present a paradox. Specifically, members of the public were more likely than doctors to believe that AI would lead to lower medical costs, whereas doctors were more likely than members of the public to express concern about the risk of data leakage. The results for these two items seem to imply that the members of the public, not the doctors, are more inclined to use AI. However, it was the doctors who gave the more affirmative responses to the actual question on the intention to use AI-driven medicine. A possible explanation for this paradox could be that the items “usefulness” and “better medical services” impact “intention to use” more than they do “medical costs” and “concern about data leakage.”

Although doctors’ total mean scores were significantly higher than those of the public, the effect size was negligible. This would be because a slight difference in the total mean score was detected by *t* test due to the large sample size.

### Limitations

Regarding the limitations of the study, one limitation concerns the possibility of sampling bias in the online survey. Because participation in the online survey was limited to individuals who could use a personal computer, smartphone, or similar device, the sample may have been biased toward the digitally literate. Moreover, as the survey was titled “Survey on AI in medicine,” the sample may have been biased toward individuals who were interested in medicine and AI. Given that people are generally more likely to express a clear opinion for or against a proposition when they are knowledgeable about the topic in question [27], a more unbiased sample may have yielded more neutral (“cannot say either way”) responses. In view of these possible biases, caution is advised when interpreting the results.

Further research is necessary to explore the relations between items. This study ascertained population trends by analyzing sample-wide responses and then comparing the responses between doctors and the public. What this approach failed to clarify was the matter of which item most affects intention to use. Accordingly, future research should explore how the responses to one item correlate with those to another. In this study, we were not able to conduct an analysis using the UTAUT model. However, since AI in medicine is now starting to be used in Japan, we would like to analyze the acceptance of AI in medicine by the UTAUT model assuming a specific system in the future study.

Since this study surveyed a large sample of 399 doctors and 600 citizens, it can be considered to have at least some validity. However, it should be noted that the questionnaire was not carried out validation.

In this study, we did not investigate the health status of the public or the duration of the professional experience of doctors. In the future, it will be necessary to conduct a survey that takes this into account.

### Conclusions

To the best of our knowledge, this is the first survey on the acceptance of AI in medicine in Japan. This study aimed to obtain detailed data on the acceptance of AI in medicine by comparing the acceptance among Japanese doctors with that among the Japanese public. An online survey was conducted, and the results were analyzed to determine sample-wide trends and trends specific to doctors and to the public.

In the 999 respondents, the results indicated that around two-thirds of the sample believed that AI would be useful in (657/999, 65.8%) and necessary to medicine (653/999, 65.4%). However, such beliefs did not directly translate to intention to use AI-driven medicine; only 447 (44.7%) of the sample expressed such a desire. The results also showed that 730 (73.1%) believed that regulatory legislation was necessary, and 734 (73.5%) were concerned about accountability, suggesting that these factors are important in terms of acceptance among doctors and the public alike. The comparison of the two groups revealed that doctors were more likely than members of the public to express intention to use AI-driven medicine (*P*<.001). This trend may be related to the responses for the items “better medical services,” “mastery,” “expectations of others,” and “interest in the topic.”

In this study, we did not analyze with the UTAUT model; however, the analysis with UTAUT should be done assuming a concrete system in the future.

## Appendix

Multimedia Appendix 1 Report on the Checklist for Reporting Results of Internet E-Surveys.

## Abbreviations

AI: artificial intelligence
CT: computed tomography
ELSI: ethical, legal, and social issue
EMR: electronic medical record
FDA: Food and Drug Administration
MHLW: Ministry of Health, Labour, and Welfare
MIC: Ministry of Internal Affairs and Communications
TAM: technology acceptance model
UTAUT: unified theory of acceptance and use of technology
