# Supplementary material for: Acceptance of the Use of Artificial Intelligence in Medicine Among Japan’s Doctors and the Public: A Questionnaire Survey
Source: JMIR Hum Factors. 2022 Mar 16;9(1):e24680. doi: 10.2196/24680 (PMC8968553; doi:10.2196/24680)
Supplement: Multimedia Appendix 1 [file humanfactors_v9i1e24680_app1.pdf]

Checklist for Reporting Results of Internet E-Surveys

Item category	Checklist item	Report in the present study
Design	Describe survey design	The target population consisted of the public and doctors in Japan. The numbers of sample were 600 members of the public and 400 doctors who registered with Rakuten Insight. The public was divided into six classes of 10 years each, with 50 men and 50 women in each class. The sample representing doctors consisted of 350 men and 50 women aged 25 years or older.
IRB (Institutional Review Board) approval and informed consent process	IRB approval	The authors did not obtained Institutional Review Board approval for this study because we did not obtain any personal or health-related information from the respondents.
	Informed consent	Respondents were told length of time to answer the questions, the purpose of the survey, and who conducted the survey on the screen just before they started to answer. Respondents were allowed to stop answering at any time until they answered all the questions. We took the completed responses as agreement of the survey by the respondents and used the responses for analysis.

Item category	Checklist item	Report in the present study
	Data protection	We collected no personal information in the present study.
Recruitment process and description of the sample having access to the questionnaire	Open survey versus closed survey	We conducted a closed survey of Rakuten Insight registrants.
	Contact mode	We did not contact with the potential respondents because we used Rakuten Insight, an internet survey service provider. They made initial contact with the respondents on the Internet.
	Advertising the survey	The survey notice was displayed on the survey panel's registrant website. The title of the questionnaire was "Survey on 'Medicine Using Artificial Intelligence'".
Survey administration	Web/E-mail	The survey was posted on the website.
	Context	The website was visited by survey panel registrants to answer surveys. Because participation in the online survey was limited to individuals who could use a personal computer, smartphone, or similar device, the sample may have been biased toward the digitally literate.

Item category	Checklist item	Report in the present study
	Mandatory/voluntary	This was a voluntary survey.
	Incentives	Respondents earned points in the survey site.
	Time/Date	The survey was conducted over three days, from November 13 to 15, 2018.
	Randomization of items or questionnaires	20 items representing a factor of acceptance was displayed randomly. Then, items on the respondents' general attributes were displayed in a fixed order.
	Adaptive questioning	We did not use adaptive questioning.
	Number of Items	The number of items per page ranged from one to seven.
	Number of screens	The number of page was seventeen.

Item category	Checklist item	Report in the present study
	Completeness check	In the items representing a factor of acceptance, there were not only positive and negative options, but also an intermediate "cannot say either way" option, so that the participants had to choose one. In addition, a system was used where the user could not proceed to the next screen unless all the questions were answered on each screen.
	Review step	In order to avoid the influence of subsequent questions, once a respondent answered a question, they could not change their answer.
Response rates	Unique site visitor	Since we used an internet survey service, it was difficult to calculate the exact response rate, so we used the sample-wide responses instead.
	View rate	
	Participation rate	
	Completion rate	

Item category	Checklist item	Report in the present study
Preventing multiple entries from the same individual	Registration	The questionnaire was displayed only once per registered survey panel member to prevent multiple entries from the same individual.
Analysis	Handling of incomplete questionnaires	We analyzed only completed questionnaires.
	Questionnaires submitted with an atypical timestamp	We did not use timestamp.
	Statistical correction	We did not use methods to adjust for the non-representative sample.
